# The effects of coagulation factors on the risk of endometriosis: a Mendelian randomization study

**DOI:** 10.1186/s12916-023-02881-z

**Published:** 2023-05-25

**Authors:** Yan Li, Hongyan Liu, Shuting Ye, Bumei Zhang, Xiaopei Li, Jiapei Yuan, Yongrui Du, Jianmei Wang, Yang Yang

**Affiliations:** 1 Department of Family Planning, The Second Hospital of Tianjin Medical University, The Province and Ministry Co-Sponsored Collaborative Innovation Center for Medical Epigenetics, Tianjin Key Laboratory of Inflammation Biology, School of Basic Medical Sciences, Tianjin Medical University, Tianjin, 300070 China; 2 State Key Laboratory of Experimental Hematology, National Clinical Research Center for Blood Diseases, Haihe Laboratory of Cell Ecosystems, Institute of Hematology and Blood Diseases Hospital, Chinese Academy of Medical Sciences and Peking Union Medical College, Tianjin, 300020 China; 3 Department of Bioinformatics, School of Basic Medical Sciences, Tianjin Medical University, Tianjin, 300070 China

**Keywords:** Two-sample Mendelian randomization, Endometriosis, Coagulation, GWAS, ADAMTS13

## Abstract

**Background:**

Endometriosis is recognized as a complex gynecological disorder that can cause severe pain and infertility, affecting 6–10% of all reproductive-aged women. Endometriosis is a condition in which endometrial tissue, which normally lines the inside of the uterus, deposits in other tissues. The etiology and pathogenesis of endometriosis remain ambiguous. Despite debates, it is generally agreed that endometriosis is a chronic inflammatory disease, and patients with endometriosis appear to be in a hypercoagulable state. The coagulation system plays important roles in hemostasis and inflammatory responses. Therefore, the purpose of this study is to use publicly available GWAS summary statistics to examine the causal relationship between coagulation factors and the risk of endometriosis.

**Methods:**

To investigate the causal relationship between coagulation factors and the risk of endometriosis, a two-sample Mendelian randomization (MR) analytic framework was used. A series of quality control procedures were followed in order to select eligible instrumental variables that were strongly associated with the exposures (vWF, ADAMTS13, aPTT, FVIII, FXI, FVII, FX, ETP, PAI-1, protein C, and plasmin). Two independent cohorts of European ancestry with endometriosis GWAS summary statistics were used: UK Biobank (4354 cases and 217,500 controls) and FinnGen (8288 cases and 68,969 controls). We conducted MR analyses separately in the UK Biobank and FinnGen, followed by a meta-analysis. The Cochran’s *Q* test, MR-Egger intercept test, and leave-one-out sensitivity analyses were used to assess the heterogeneities, horizontal pleiotropy, and stabilities of SNPs in endometriosis.

**Results:**

Our two-sample MR analysis of 11 coagulation factors in the UK Biobank suggested a reliable causal effect of genetically predicted plasma ADAMTS13 level on decreased endometriosis risk. A negative causal effect of ADAMTS13 and a positive causal effect of vWF on endometriosis were observed in the FinnGen. In the meta-analysis, the causal associations remained significant with a strong effect size. The MR analyses also identified potential causal effects of ADAMTS13 and vWF on different sub-phenotypes of endometrioses.

**Conclusions:**

Our MR analysis based on GWAS data from large-scale population studies demonstrated the causal associations between ADAMTS13/vWF and the risk of endometriosis. These findings suggest that these coagulation factors are involved in the development of endometriosis and may represent potential therapeutic targets for the management of this complex disease.

**Supplementary Information:**

The online version contains supplementary material available at 10.1186/s12916-023-02881-z.

## Background

Endometriosis is defined as the deposit and growth of endometrial tissue that normally lines the inside of the uterus outside the uterine cavity [1]. Women who have endometriosis are more likely to experience dysmenorrhea, pelvic pain, and even infertility or difficulty conceiving. Endometriosis is a common and complex disorder that affects up to 6–10% of all reproductive-aged women [2]. Although many factors, including hormones, inflammation, genetic factors, epigenetic factors, and environmental factors, are thought to contribute to the development of endometriosis, the etiology and pathogenesis of endometriosis have not been completely elucidated [3, 4].

Among the hypotheses that have been proposed to explain the pathogenesis of endometriosis, retrograde menstruation, also known as Sampson’s theory, is the most widely accepted [4, 5]. According to the model of retrograde menstruation, endometrial tissues are shed through the fallopian tubes into the pelvic cavity during menstruation, resulting in the formation of ectopic endometriotic lesions on the peritoneal tissue or pelvic organs. The ectopic debris could be cleared by the immune system in healthy women, whereas the refluxed endometrial fragments might evade the immune surveillance system in endometriosis patients [6–8]. Defective immune surveillance is thought to play a role in the implantation and growth of ectopic endometrial tissue [6]. Endometriosis is also considered as a chronic inflammatory disease, owing to the presence of ectopic endometrial fragments, which cause an increase in proinflammatory factors and chemotactic cytokines [9–11]. Furthermore, angiogenesis is required to replenish the supply of nutrients and oxygen for the growth and survival of endometriotic lesions [12, 13]. Coagulation cascades have been implicated in both inflammatory responses and angiogenesis [12, 14–16]. Several epidemiological observational studies have found that patients with endometriosis are hypercoagulable and hyperfibrinolytic [17, 18]. Plasma fibrinogen, d-dimer, and plasminogen activator inhibitor levels are higher in women with endometriosis when compared to healthy controls while thrombin time and activated partial thromboplastin time decrease [19]. Adenomyosis, a condition characterized by endometrial tissue growth within the uterine musculature, shares numerous common symptoms with endometriosis, including pelvic pain and heavy menstrual bleeding [20]. Harmsen et al. have reported the increased levels of von Willebrand factor in ectopic endometrium of adenomyosis patients which are associated with the role of angiogenesis in adenomyosis [21]. Although several observational studies have been conducted to explore the relationship between coagulation cascades and endometriosis, the causal associations between coagulation factors and endometriosis remain unclear. The presence of residual confounding and potential reverse causality issues in conventional observational studies poses significant challenges in accurately measuring the causal effect of specific coagulation factor on the risk of endometriosis. Residual confounding occurs as a result of inadequate adjustment for confounding variables, as measuring a confounder may not fully characterize it. In addition, the association between the exposure and outcome may occur due to reverse causality, a phenomenon in which the outcome precedes and causes the exposure, rather than the exposure causing the outcome.

As an emerging method, Mendelian randomization (MR) is a novel statistical method that examines the causal relationship between the exposure and outcome by using genetic variants as instrumental variables for the exposure of interest [22, 23]. Because genetic variants are randomly allocated during gamete formation and conception, MR analysis could reduce confounding bias and reverse causality [23]. A two-sample MR analysis was carried out in this study to investigate the causal effects of coagulation factors on endometriosis. There were 11 coagulation factors incorporated as the exposures, including vWF (von Willebrand factor), ADAMTS13 (A disintegrin and metalloproteinase with thrombospondin motifs 13), aPTT (activated partial thromboplastin time), FVIII (factor VIII), FXI (factor XI), FVII (factor VII), FX (factor X), ETP (endogenous thrombin potential), PAI-1 (plasminogen activator inhibitor-1), protein C, and plasmin. We leveraged summary-level GWAS data from two independent large-scale cohorts of European ancestry, including the UK Biobank and FinnGen cohorts, to estimate a putative causal association of a specific coagulation factor with the risk of endometriosis.

## Methods

### Study design

**Fig. 1:**
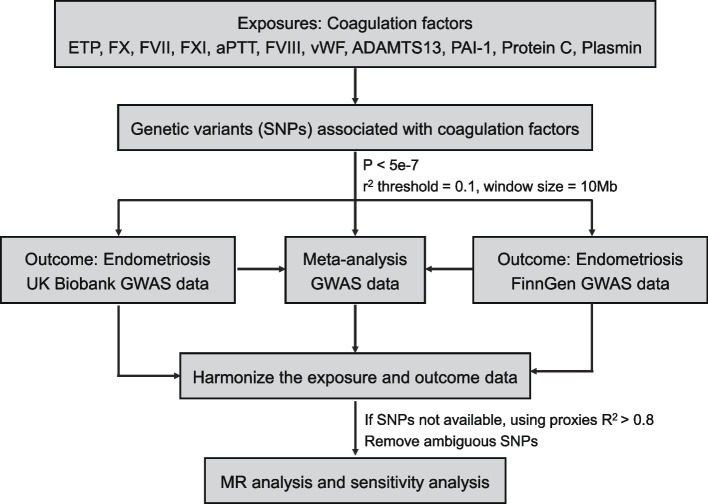
Overall design of the MR analysis framework in this study. A flow chart depicts how the MR analysis was conducted step by step in this study

Three critical assumptions must be met in the MR analysis. The first assumption is that the genetic variables should be significantly related to the exposure, the second assumption is that genetic variants extracted as instrumental variables for the exposure are not related to other confounding factors, and the third assumption is that genetic variants influence the outcome solely through their effects on the exposure (i.e., no horizontal pleiotropic effect) [24]. Figure 1 depicts the overall design of this study. We began by selecting 11 coagulation factors based on publicly available GWAS data. Based on the GWAS summary statistics, we selected instrumental variables for each coagulation factor. Then, using summary-level GWAS data of endometriosis from two independent cohorts, including the UK Biobank and FinnGen, we conducted two-sample MR analyses separately to estimate the causal effects of coagulation factors on endometriosis. To confirm the potential causal effects of coagulation factors, we further meta-analyzed endometriosis GWAS summary statistics from the UK Biobank and FinnGen. Finally, MR analyses were also performed to estimate the causal associations of coagulation factors with the risk of various sub-phenotypes of endometrioses, including endometriosis of the intestine, ovary, pelvic peritoneum, fallopian tube, uterus, rectovaginal septum, and vagina.

### Endometriosis GWAS summary statistics

To obtain a reliable conclusion of the causal relationships between coagulation factors and the risk of endometriosis, we have conducted a systematic analysis of endometriosis GWAS summary-level data collected from two large-scale cohorts, including the UK Biobank and FinnGen. The GWAS summary statistics for endometriosis among individuals of European ancestry in the UK Biobank were procured from the Pan-UK Biobank website (https://pan.ukbb.broadinstitute.org/) via a phenotype description search for “endometriosis” [25]. Correspondingly, the FinnGen cohort’s endometriosis GWAS summary statistics were accessible via the R package TwoSampleMR (v 0.5.6) [26] using the GWAS ID “finn-b-N14_ENDOMETRIOSIS” as documented in the IEU OpenGWAS database (https://gwas.mrcieu.ac.uk/) [27]. In the UK Biobank, the diagnosis of endometriosis was defined by N80 in the International Classification of Diseases, 10th Revision (ICD-10). The GWASs for endometriosis from the UK Biobank of European ancestry were conducted on 4354 cases and 217,500 female controls. In FinnGen, endometriosis is defined by N80 in ICD-10, 617 in ICD-9, and 6253 in ICD-8. The GWAS summary statistics for endometriosis from FinnGen included 8288 cases and 68,969 controls. In addition, we also curated summary-level GWAS data from the FinnGen cohort for various sub-phenotypes of endometrioses, including endometriosis of the uterus (2372 cases, 68,969 controls), endometriosis of the ovary (3231 cases, 68,969 controls), endometriosis of the fallopian tube (116 cases, 68,969 controls), endometriosis of the pelvic peritoneum (2953 cases, 68,969 controls), endometriosis of the rectovaginal septum and vagina (1360 cases, 68,969 controls), and endometriosis of the intestine (177 cases, 68,969 controls).

### Genetic instrumental variable selection

We used instrumental variables to investigate the causal associations between coagulation factors and endometriosis. We searched for GWASs of coagulation factors in European populations to curate genetic variants associated with coagulation factors. vWF, ADAMTS13, aPTT, FVII, FXI, FVII, FX, ETP, PAI-1, protein C, and plasmin were chosen as the examined coagulation factors with available genome-wide significant SNPs [28–36] (Additional file 1: Table S1). Then, for each coagulation factor, we went through a stringent quality control procedure to select eligible instrumental variables for each coagulation factor. First, we selected SNPs associated with specific coagulation factors at genome-wide significance (*P* < 5e − 7) as candidate instrumental variables for further MR analysis. Second, to ensure the instrumental variables for each exposure phenotype are independent, we used the linkage disequilibrium (LD)-based clumping to remove SNPs in strong LD (*r*^2^ threshold = 0.1, window size = 10 Mb). The clumping step was carried out based on the European reference panel of the 1000 Genomes Project, which was used to estimate LD between SNPs.

For SNPs that were not present in the endometriosis GWAS data, we used the LDlink tools to search for the most correlated proxy SNPs using the 1000 Genomes of European population data (*r*^2^ > 0.8) [37]. We also discarded SNPs with non-concordant alleles and palindromic SNPs with ambiguous strands that could not be corrected when harmonizing the exposure data and outcome data. These stringently filtered SNPs were used as the instrumental variables for subsequent MR analyses. To determine whether there was a weak instrumental variable bias, we calculated *F*-statistics to quantify the strength of instrumental variables, where *F*-statistics larger than 10 indicates a low possibility of weak instrumental variable bias [38, 39] (Additional file 1: Table S1). All the instrumental variable selection and quality control steps are performed using the R package TwoSampleMR (v 0.5.6) [26].

### Statistical power calculation

We sought to assess the statistical power of our MR analyses through the use of an online web tool specialized for binary outcomes (https://sb452.shinyapps.io/power) [40]. The assessment of statistical power for MR analyses was based on several parameters, including the total sample size, the significance level of 0.05, the proportion of variance (*R*^2^) in the exposure explained by instrumental variables, and the ratio of cases to controls.

### Mendelian randomization estimates

We combined the summary statistics (*β* coefficients and standard errors) to estimate the causal associations between 11 coagulation factors and endometriosis separately using different MR methods. The MR analyses were first performed separately in the UK Biobank and FinnGen cohorts. Three MR methods based on different assumptions were applied: inverse variance weighting (IVW), weighted mean (WM), and MR-Egger regression. The IVW method was utilized as the main statistical model. There are fixed effects and random effects IVW methods available. We first calculated the causal estimates using the fixed effects IVW methods by meta-analyzing Wald ratio estimates for each instrumental variable. If significant heterogeneity (*P* < 0.05) is observed, the random effects IVW method is added. In addition, we also conducted MR analyses based on the meta-analyzed summary statistics which are combined from the UK Biobank and FinnGen using the METAL tool [41].

Causal estimates from MR analyses can only be interpreted reliably if the three critical assumptions are met. Heterogeneity in causal estimates among instrumental variables indicates a potential violation of the assumptions of MR analysis [42]. The Cochran’s *Q* test was used to examine the heterogeneity in causal estimates, and we used both the causal estimates of fixed effects IVW method and MR-Egger regression to detect heterogeneity. The heterogeneities were quantified using Cochran’s *Q* statistics and a *P*-value smaller than 0.05 was considered significant heterogeneity. To assess the potential pleiotropic effects of instrumental variables, the MR-Egger regression was used. The directional horizontal pleiotropy in the causal estimates may be indicated by the intercept term in MR-Egger regression. Additionally, we performed a leave-one-out analysis where we excluded each SNP in turn and then ran MR analysis on the remaining SNPs in order to detect potentially outlying instrumental variables [26]. The Steiger test of directionality is also conducted to assess the causal relationship between the exposure and outcome. All MR analyses were performed using the R package TwoSampleMR (v 0.5.6) [26].

## Results

### Selection of instrumental variables

We systematically curated genome-wide significant SNPs associated with 11 coagulation factors (vWF, ADAMTS13, aPTT, FVIII, FXI, FVII, FX, ETP, PAI-1, protein C, and plasmin) from different GWAS results through literature searching to examine the potential causal effects of these coagulation factors on the risk of endometriosis [28–36] (Additional file 1: Table S1). These coagulation factors could be categorized into five groups, including platelet adhesion (vWF and ADAMTS13), intrinsic pathway (FXI, aPTT, and FVIII), extrinsic pathway (FVII), common pathways (ETP and FX), and fibrin clot dissociation (PAI-1, protein C, and plasmin). We first kept the SNPs that were significantly associated with each exposure phenotype in the corresponding GWAS study (*P* < 5e − 7). Then, we used LD-based clumping to obtain the LD-independent SNPs for the exposure (*r*^2^ threshold = 0.1, window size = 10 Mb). It is critical that the effect of an SNP on the exposure and the effect of that on the outcome are both attributed to the same allele. In the harmonizing process, ambiguous SNPs with non-concordant alleles and palindromic SNPs with ambiguous strands that cannot be corrected were discarded. Therefore, the number of SNPs chosen as instrumental variables for the exposure in subsequent two-sample MR analyses would eventually be equal to or less than that listed in Additional file 1: Table S1. To assess the strength of each instrumental variable, we calculated the *F*-statistics for each instrument-exposure association. In our study, the *F*-statistics were much greater than 10, indicating that those SNPs were strong instrumental variables (Additional file 1: Table S1). Moreover, we have calculated the statistical power for every exposure in each cohort. Notably, the results indicated that the statistical power ranged from 80% to 100% for all coagulation factors, thereby affirming the robustness of our subsequent MR analyses (Additional file 1: Table S1).

### Causal effects of coagulation factors on endometriosis

**Fig. 2:**
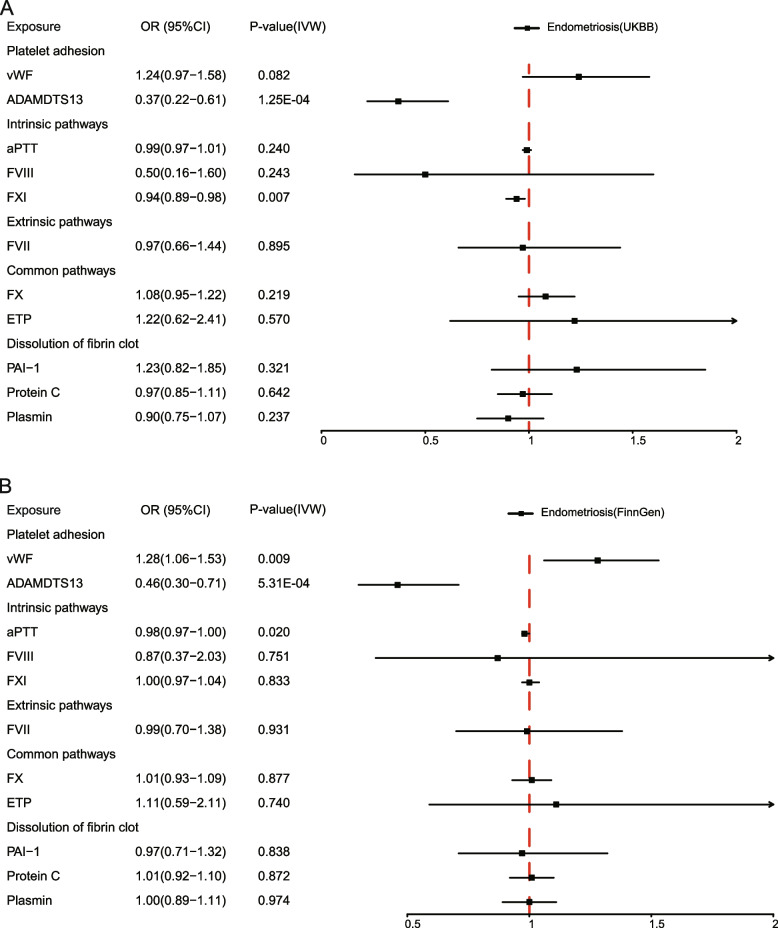
Causal estimates of 11 coagulation factors on endometriosis by MR analysis. **A** Forest plots showing causal estimates of 11 coagulation factors on endometriosis estimated in the UK Biobank of European ancestry. **B** Forest plots showing causal effects of 11 coagulation factors on endometriosis estimated in FinnGen. The odds ratio (OR) was estimated using the fixed effect IVW method. The horizontal bars represent 95% confidence intervals (CI)

Based on the GWAS summary statistics for endometriosis in the UK Biobank of European ancestry, which included 4354 cases and 217,500 controls, we performed MR analyses to estimate the causal effects of 11 coagulation factors on the risk of endometriosis. The MR estimates from different methods were shown in Additional file 1: Table S2. The findings demonstrated that the genetically predicted plasma ADAMTS13 level is causally associated with a decreased risk of endometriosis (IVW: OR = 0.37, 95%CI: 0.22–0.61, *P* = 1.25e − 4; WM: OR = 0.41, 95%CI: 0.23–0.72, *P* = 2.05e − 3) (Fig. 2A, Additional file 1: Table S2, Additional file 2: Fig. S1). Notably, after accounting for multiple comparisons across 11 coagulation factors, the negative causal effects of plasma ADAMTS13 level on endometriosis remained significant (IVW: *P*_adjusted_ = 1.38e − 3). Furthermore, we discovered a mild negative causal relationship between genetically predicted FXI levels and endometriosis (IVW: OR = 0.94, 95%CI: 0.89–0.98, *P* = 7.08e − 3; WM: OR = 0.95, 95%CI: 0.89–1.00, *P* = 0.059) (Fig. 2A, Additional file 1: Table S2, Additional file 2: Fig. S1). However, other coagulation factors (vWF, aPTT, FVIII, FVII, FX, ETP, PAI-1, protein C, and plasmin) had no significant causal effect on endometriosis (Fig. 2A, Additional file 1: Table S2, Additional file 2: Fig. S1). Heterogeneity tests revealed heterogeneity in endometriosis for three coagulation factors, vWF (IVW: Cochran’s *Q* = 20.35, *P*_heterogeneity_ = 0.041), aPTT (IVW: Cochran’s *Q* = 16.57, *P*_heterogeneity_ = 0.011), and FVIII (IVW: Cochran’s *Q* = 11.80,* P*_heterogeneity_ = 0.003) (Additional file 1: Table S2). Additional MR analyses using the random effects IVW method yielded causal effect estimates that were consistent with those estimated using the fixed effects IVW method (Additional file 1: Table S2). In the MR-Egger intercept test, we detected no significant evidence of horizontal pleiotropy (*P*_pleiotropy_ > 0.05) (Additional file 1: Table S2). Further leave-one-out analyses were carried out to ascertain potential outliers in the instrumental variable estimation of ADATMS13 and FXI causal effects on the risk of endometriosis (Additional file 1: Table S3, Additional file 2: Fig. S2). Through the Steiger test of directionality, the results corroborated the negative causal effects of ADAMTS13 and FXI on the risk of endometriosis (Additional file 1: Table S2). As a result of the MR analyses in the UK Biobank cohort, we were able to draw a robust conclusion that the genetically predicted plasma ADAMTS13 levels are causally associated with a decreased risk of endometriosis, and the association between FXI and the decreased risk of endometriosis is likely to be causal.

As a replication analysis, we performed MR analyses based on the GWAS summary statistics for endometriosis in FinnGen (8288 cases and 68,969 controls). The findings highlighted that the negative causal effects of the genetically predicted plasma ADAMTS13 level on the risk of endometriosis remained significant with a large effect size (IVW: OR = 0.46, 95%CI: 0.30–0.71, *P* = 5.31e − 4; WM: OR = 0.53, 95%CI: 0.33–0.85, *P* = 0.009), which was consistent with the findings from the UK Biobank (Fig. 2B, Additional file 1: Table S4, Additional file 2: Fig. S3). After multiple test correction, the causal association estimated using fixed effects IVW method remained significant (IVW: *P*_adjusted_ = 5.8e − 3). Despite the presence of heterogeneity in the causal estimates for ADAMTS13 on endometriosis in FinnGen (IVW: Cochran’s *Q* = 11.91,* P*_heterogeneity_ = 0.003), the causal effects estimated using the random effects IVW method remained borderline significantly with a strong effect size (IVW: OR = 0.46, 95%CI: 0.16–0.1.34, *P* = 0.056) (Additional file 1: Table S4). The results also showed that the genetically predicted plasma vWF level was positively causally associated with the risk of endometriosis (IVW: OR = 1.28, 95%CI: 1.06–1.53, *P* = 0.009; WM: OR = 1.33, 95%CI: 1.08–1.62, *P* = 0.006), although the effect may not remain significant after adjusting for multiple comparisons (Fig. 2B, Additional file 1: Table S4, Additional file 2: Fig. S3). Conversely, the significant negative causal relationship between FXI and endometriosis observed in the UK Biobank was not replicated in FinnGen (Fig. 2B, Additional file 1: Table S3, Additional file 2: Fig. S3). We observed no obvious horizontal pleiotropy in the MR-Egger intercept test and no potentially influential instrumental variable in the leave-one-out analysis for ADAMTS13 and vWF (Additional file 1: Table S4 and S5, Additional file 2: Fig. S4). The directionality of their causal effects was also confirmed using the Steiger test (Additional file 1: Table S4). In conclusion, our FinnGen cohort results suggest that ADAMTS13 levels are causally associated with a decreased risk of endometriosis, and the positive association observed between vWF and the risk of endometriosis is likely to be causal.

**Fig. 3:**
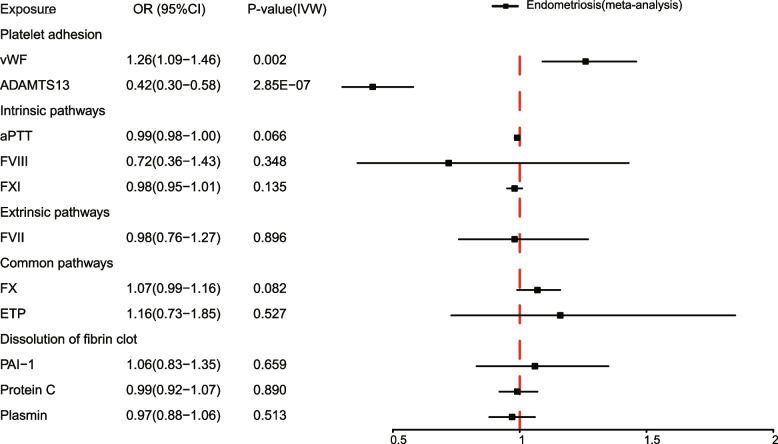
Causal estimates of 11 coagulation factors on endometriosis in a meta-analysis. Forest plots showing causal estimates of 11 coagulation factors on endometriosis in a meta-analysis of UK Biobank and FinnGen. The odds ratio (OR) was estimated using the fixed effect IVW method. The horizontal bars represent 95% confidence intervals (CI)

With the purpose of verifying the causal effects of coagulation factors on endometriosis, we meta-analyzed the GWAS summary statistics obtained from the UK Biobank and FinnGen, thereby enhancing the sample size and statistical power. Subsequent MR analyses were carried out using the meta-analyzed GWAS summary statistics for endometriosis. The results supported the strong causal effect of ADAMTS13 on the decreased risk of endometriosis (IVW: OR = 0.42, 95%CI: 0.30–0.58, *P* = 2.85e − 7; WM: OR = 0.44, 95%CI: 0.30–0.66, *P* = 5.76e − 5) (Fig. 3, Additional file 1: Table S6, Additional file 2: Fig. S5). Notably, heterogeneity in causal estimates of ADAMTS13 was detected by the heterogeneity test (IVW: Cochran’s *Q* = 13.23, *P*_heterogeneity_ = 0.004), necessitating use of the random effects IVW method to evaluate the causal association. The result from random effects IVW analysis confirmed the strong negative causal link between ADAMTS13 and endometriosis (Additional file 1: Table S6). The significant MR result of vWF on the risk of endometriosis was also observed (IVW: OR = 1.26, 95%CI: 1.09–1.46, *P* = 0.002; WM: OR = 1.29, 95%CI: 1.10–1.51, *P* = 0.002) (Fig. 3, Additional file 1: Table S6, Additional file 2: Fig. S5). Moreover, the absence of potentially influential instrumental variables was ascertained by leave-one-out analysis (Additional file 1: Table S7, Additional file 2: Fig. S6), and the Steiger test validated the directionality of the causal effects on the risk of endometriosis (Additional file 1: Table S6). Summarizing the findings from the meta-analysis, we could conclude that the genetically predicted plasma ADAMTS13 levels have a negative causal effect on the risk of endometriosis, suggesting that ADAMTS13 serves as a protective factor for endometriosis. Conversely, the genetically predicted plasma vWF levels are positively associated with the risk of endometriosis, indicating vWF function as a risk factor for the development of endometriosis.

### Causal effects of coagulation factors on different sub-phenotypes of endometrioses

Depending on the location and growth of ectopic endometriotic lesions, endometriosis could be categorized. The precise sub-phenotypes of endometriosis experienced by patients may have an impact on both their symptoms as well as their chance of infertility. Endometrioses of the intestine, ovary, pelvic peritoneum, uterus, fallopian tube, and rectovaginal vaginal regions were among the five sub-phenotypes of endometrioses diagnosed in the FinnGen cohort. The GWAS summary statistics of various sub-phenotypes of endometrioses were also available in the FinnGen cohort. The number of patients ranged from 116 in endometriosis of the fallopian tube to 3231 in endometriosis of the ovary. Some patients might have more than one sub-phenotype of endometriosis because there was an overlap between different sub-phenotypes.

**Fig. 4:**
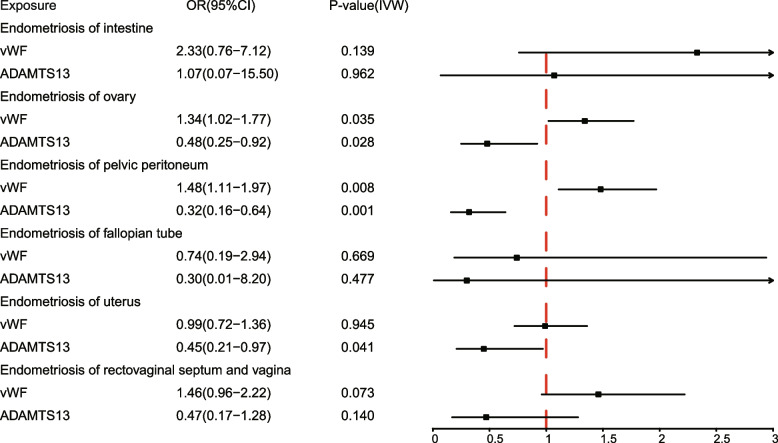
Causal estimates of vWF and ADAMTS13 on different sub-phenotypes of endometrioses. Forest plots depicting causal estimates of vWF and ADAMTS13 on different sub-phenotypes of endometrioses in FinnGen, including endometriosis of intestine, endometriosis of ovary, endometriosis of pelvic peritoneum, endometriosis of uterus, endometriosis of the fallopian tube, and endometriosis of the rectovaginal septum and vagina. The odds ratio (OR) was estimated using the fixed effect IVW method. The horizontal bars represent 95% confidence intervals (CI). Significant *P* values are highlighted in red

We employed MR analyses to further investigate the causal effects of genetically predicted plasma levels of ADAMTS13 and vWF on the risk of various sub-phenotypes of endometrioses. The findings demonstrated that ADAMTS13 is negatively causally associated with the risk of endometriosis of the ovary (IVW: OR = 0.48, 95%CI: 0.25–0.92, *P* = 0.028; WM: OR = 0.58, 95%CI: 0.2–81.20, *P* = 0.140), endometriosis of the pelvic peritoneum (IVW: OR = 0.32, 95%CI: 0.16–0.64, *P* = 0.001; WM: OR = 0.40, 95%CI: 0.19–0.85, *P* = 0.017), and endometriosis of the uterus (IVW: OR = 0.45, 95%CI: 0.21–0.97, *P* = 0.041; WM: OR = 0.44, 95%CI: 0.20–0.99, *P* = 0.048) (Fig. 4, Additional file 1: Table S8). In addition, ADAMTS13 had a negative but not statistically significant causal effect on endometriosis of the rectovaginal septum and vagina, and there was no evidence of a causal effect of ADAMTS13 on endometriosis of the intestine (Fig. 4, Additional file 1: Table S8). As heterogeneity was detected, we conducted a random effects IVW analysis to validate the findings (Additional file 1: Table S8). From the random effects IVW analysis, the causal estimates of ADAMTS13 on endometriosis of the uterus remained borderline significant (IVW: OR = 0.45, 95%CI: 0.20-–1.02, *P* = 0.051), while the causal estimates for endometrioses of the ovary (IVW: OR = 0.48, 95%CI: 0.13–1.79, *P* = 0.274) and pelvic peritoneum (IVW: OR = 0.32, 95%CI:0.08–1.32, *P* = 0.116) attenuated towards non-significance (Additional file 1: Table S8). Meanwhile, the significant causal estimates of vWF were also observed for endometriosis of the ovary (IVW: OR = 1.34, 95%CI: 1.02–1.77, *P* = 0.035; WM: OR = 1.37, 95%CI: 1.03–1.81, *P* = 0.028) and endometriosis of the pelvic peritoneum (IVW: OR = 1.48, 95%CI: 1.11–1.97, *P* = 0.008; WM: OR = 1.53, 95%CI: 1.13–2.08, *P* = 0.006) (Fig. 4, Additional file 1: Table S8). In summary, the evidence suggests that ADAMTS13 may have a negative causal relationship with endometriosis of the ovary, pelvic peritoneum, and uterus, while vWF may have a positive causal relationship with endometriosis of the ovary and pelvic peritoneum.

In addition, we noticed that the ratios of cases to controls significantly varied across sub-phenotypes, ranging from 1/594 for endometriosis of the fallopian tube to 1/21 for endometriosis of the ovary. Such disparity may impede the statistical power of a MR study, prompting the need to evaluate the statistical power. To establish the validity of the results, we additionally calculated the statistical power for the MR analysis in each sub-phenotype cohort. The statistical power was merely about 14% and 20% for sub-phenotypes of the fallopian tube and intestine, respectively (Additional file 1: Table S8). Therefore, we should draw our conclusions with cautions for these two sub-phenotypes. In contrast, the statistical power for the other four sub-phenotypes, including endometriosis of the uterus, ovary, pelvic peritoneum, and rectovaginal septum and vagina ranged between 80% and 1, thus affirming the robustness of the MR results of these sub-phenotypes (Additional file 1: Table S8).

In addition, the condition of endometriosis of the uterus, also referred to as adenomyosis, has been categorized as a separate disease, despite its classification as a form of endometriosis in ICD-10. Several studies have suggested that endometriosis and adenomyosis share similar pathophysiology, specifically related to somatic epithelial mutations and epigenetic abnormalities. In order to determine the potential effects of incorporating endometriosis of the uterus in our MR study, we employed LDSC to examine the genetic correlations between adenomyosis and other sub-phenotypes [43, 44]. The results indicate strong genetic correlations, ranging from 0.67 to 0.93, indicating a shared genetic architecture and pathophysiological mechanisms between adenomyosis and endometriosis (Additional file 1: Table S9). These findings suggest that the inclusion of adenomyosis is unlikely to significantly impact the causal estimation of coagulation factors on the risk of endometriosis.

## Discussion

Utilizing summary statistics from two large-scale GWASs of European ancestry including UK Biobank and FinnGen, we investigated the causal effects of 11 coagulation factors on the risk of endometriosis, employing a unified MR framework to analyze GWAS data. Our results indicate that genetically predicted plasma ADAMTS13 levels were inversely associated with endometriosis, while genetically predicted plasma vWF levels demonstrated a positive causal association with endometriosis, as confirmed in the meta-analysis combining the cohorts. Furthermore, MR analyses also revealed the causal associations in different sub-phenotypes of endometrioses that are categorized by ectopic location. These findings have significant implications for the development of endometriosis prevention strategies and treatment methods. For example, the findings underscore the significance of monitoring the ADAMTS13 plasma levels in individuals diagnosed with endometriosis. Furthermore, the results also provide a potential therapeutic approach that entails regulating the ADAMTS13 plasma level, thereby enabling the management and prevention of endometriosis progression and recurrence.

Although several factors involved in the development of endometriosis have been uncovered, the precise etiology and pathogenesis of endometriosis remain obscure, and its treatment remains controversial [3, 4]. A thorough understanding of endometriosis is required for the development of effective preventative and treatment strategies. Sampson proposed the retrograde menstruation theory, which states that menstrual blood containing endometrial cells retrograde through fallopian tubes into the pelvic cavity instead of out of the body, leading to the formation of ectopic endometriotic lesions [45]. Although Sampson’s theory is the most widely accepted, several alternative hypotheses have been put forth, such as the theories of stem cell origin and altered immunity [46, 47]. Endometriosis is considered as a consequence of a complex interplay of genetic, anatomical, environmental, and immunologic factors [1–3]. Despite contradicting accounts regarding the origin of endometriosis, it is generally accepted that endometriosis is associated with a local inflammatory response, and that vascularization at the site of endometriotic invasion plays a crucial role in the development of the lesions [48]. Notably, the coagulation system has been acknowledged as playing critical roles in modulating both inflammatory responses and angiogenesis [12, 14–16]. Recently, Li et al. have reported that the fibrinogen alpha chain could promote the migration and invasion of endometrial cells and promote angiogenesis in endometriosis [49–52]. Heavy menstrual bleeding (HMB) is a prevalent clinical symptom of endometriosis. Studies have raised the possibility of an imbalance in coagulation factors playing a role in HMB in patients with endometriosis. Research has noted that women with endometriosis exhibit a hypercoagulable status characterized by elevated levels of specific coagulation factors, such as fibrinogen and vWF [17–19, 53, 54]. These elevated factors may contribute to HMB by promoting the formation of blood clots. As such, an imbalanced coagulation system may represent a plausible etiologic mechanism behind HMB in endometriosis. Despite the growing interest regarding the involvement of coagulation factors in the pathogenesis of endometriosis, the causal roles of these factors in the development of endometriosis remain uncertain.

This is the first study to investigate the causal relationships between coagulation factors and the risk of endometriosis utilizing MR analyses on large-scale population cohorts, which provided unconfounded causal estimates. The findings highlighted that the plasma ADAMTS13 levels have a negative causal effect on endometriosis, whereas the plasma vWF levels have a positive causal effect on endometriosis. In other words, ADAMTS13 is found to have a protective effect associated with endometriosis, while vWF is characterized as a risk factor for the development of the condition. The multimeric glycoprotein vWF is stored in the Weibel-Palade bodies and α-granules of platelets, awaiting release upon stimulation. Its primary function involves the formation of a bridge between surface receptors on platelets and the endothelium, allowing for platelet recruitment following an injury [55]. ADAMTS13 is a multidomain metalloprotease that is predominantly synthesized in the liver by hepatic stellate cells, and its primary role is to regulate thrombogenesis by cleaving hyperactive ultra-large multimers of vWFs into less active, smaller fragments [56]. Given the vWF-cleaving function of ADAMTS13, the biological functions of ADAMTS13 and vWF are closely related. The thrombotic thrombocytopenic purpua (TTP) that arises in people with severe ADAMTS13 deficiency has highlighted the relevance of ADAMTS13 function [57, 58]. ADAMTS13 deficiency may lead to the accumulation of vWF multimers, which causes intravascular platelet aggregation and microthrombosis, resulting in TTP. Aside from the well-established role in hemostasis, the balance between ADAMTS13 and vWF has been linked to a variety of diseases, such as systemic inflammation, pancreatitis, and multiple sclerosis [59–61]. The biosynthesis and secretion of ADAMTS13 from vascular endothelial cells have raised the interests in the role of ADAMTS13 in angiogenesis [62–64]. The balance between ADAMTS13 and vWF is crucial for controlling angiogenesis, as demonstrated by numerous studies [63]. In addition, Xiao et al. have recently demonstrated the proteolytically active ADAMTS13 is expressed in the human placental tissues and has a role in trophoblast cell proliferation, migration, invasion, and tube formation [65]. Overall, the balance between ADAMTS13 and vWF not only regulates hemostasis, but also exerts a role in inflammation modulation, regulating angiogenesis, and tissue remodeling. Our findings of this MR study confirmed the causal roles of ADAMTS13 and vWF on endometriosis. Although the UK Biobank and FinnGen cohorts were utilized, there remains a need for independent validation of these causal relationships. Furthermore, given the potential pathophysiology of endometriosis, a more comprehensive understanding of the molecular mechanisms and action of these coagulation factors in endometriosis pathogenesis requires additional experimental validation.

There are several strengths in this study. First, because it is based on the fact that genetic variants are randomly allocated during gamete formation and conception, the results of MR analysis are less susceptible to confounding bias and reverse causality [23]. Second, we employed separate samples for the exposures (coagulation factors) and the outcome (endometriosis) data to ensure two-sample MR analyses, which avoid inflating the bias of weak instrumental variables. Third, we incorporated two independent large-scale cohorts for MR analyses, followed by a meta-analysis, so that a sufficiently enough sample size of the outcome could assure the generalizability of causal associations. In addition, the consistent causal effect estimates of ADAMTS13 on endometriosis among the UK Biobank, FinnGen, and the meta-analysis alleviated concerns on false-positive results. Fourth, we employed multiple supplementary analyses, such as heterogeneity, pleiotropy, and leave-one-out sensitivity analyses, to verify the viability of the assumptions regarding the instrumental variables.

Nonetheless, several limitations also need to be acknowledged. First, the number of instrumental variables for each coagulation factor, as outlined in Additional file 1: Table S1 ranged from three to thirteen. Furthermore, some instrumental variables will be discarded during MR analyses when harmonizing the exposure and outcome data. These limitations suggest that the final MR estimates may be subject to influence from the limited number of instrumental variables. Nevertheless, the statistical power calculations for each coagulation factor within each cohort indicate that adequate power was achieved, with estimated statistical power ranging from 80% to 1. Therefore, despite the potential limitations, the results presented in this study remain sufficiently powered to draw robust conclusions. Second, only genome-wide significant SNPs for different coagulation factors were available in the exposure GWAS data, preventing us from performing bi-directional MR analyses. Third, in the context of endometriosis, a female-specific condition, it is noteworthy that existing GWASs examining diverse coagulation factors have been conducted on a sex-combined bias. As two-sample MR necessitates consistency in the underlying population for both sample sets, it is important to consider potential discrepancies with regard to genetic estimates of coagulation factors in females versus males, which may introduce bias into our MR findings. Fourth, because this study was limited to people of European ancestry, the findings may not be generalizable to other populations. More studies into the causal associations between coagulation factors and endometriosis in other populations are needed.

## Conclusions

To the best of our knowledge, this is the first MR study to examine the causal associations between coagulation factors and the risk of endometriosis in the European population. The findings convincingly support the causal associations between ADAMTS13/vWF and the risk of endometriosis. This study contributes to a better understanding of the involvement of coagulation cascades in the development of endometriosis. These findings may have important implications for endometriosis prevention and treatment strategies.

## Supplementary Information

**Additional file 1: Table S1.** Selected instrumental variables for coagulation factors in this study. **Table S2.** Summary statistics of the causal estimates of coagulation factors on endometriosis in UK Biobank. **Table S3.** The results of leave-one-out analyses for endometriosis in UK Biobank. **Table S4.** Summary statistics of the causal estimates of coagulation factors on endometriosis in FinnGen. **Table S5.** The results of leave-one-out analyses for endometriosis in FinnGen. **Table S6.** Summary statistics of the causal estimates of coagulation factors on endometriosis in the meta-analysis. **Table S7.** The results of leave-one-out analyses for endometriosis in the meta-analysis. **Table S8.** Summary statistics of the causal estimates of vWF and ADAMTS13 on different sub-phenotypes of endometrioses. **Table S9.** Genetic correlations between endometriosis of uterus (adenomyosis) and other sub-phenotypes.

**Additional file 2: Fig. S1.** Scatter plots for MR analyses of the causal effect of 11 coagulation factors on endometriosis in UK Biobank. **Fig. S2.** Plots of leave-one-out analyses for the causal associations in UK Biobank. **Fig. S3.** Scatter plots for MR analyses of the causal effect of 11 coagulation factors on endometriosis in FinnGen. **Fig. S4.** Plots of leave-one-out analyses for the causal associations in FinnGen. **Fig. S5.** Scatter plots for MR analyses of the causal effect of ADAMTS13 and vWF on endometriosis in a meta-analysis. **Fig. S6.** Plots of leave-one-out analyses for the causal associations in the meta-analysis.

## Data Availability

The GWAS summary statistics for coagulation factors are available in the GWAS Catalog or the published article and its supplementary files. The GWAS summary statistics for endometriosis are available on the Neale lab Pan-UK Biobank website (https://pan.ukbb.broadinstitute.org/) for the UKBB cohort and the IEU GWAS database (https://gwas.mrcieu.ac.uk/) for FinnGen [25, 27].

## Abbreviations

CI: Confidence interval
GWAS: Genome-wide association study
IVW: Inverse variance weighting
LD: Linkage disequilibrium
MR: Mendelian randomization
OR: Odds ratio
RCTs: Randomized controlled trials
SNP: Single nucleotide polymorphism
WM: Weighted mean
