# Supplementary material for: The effects of coagulation factors on the risk of endometriosis: a Mendelian randomization study
Source: BMC Med. 2023 May 25;21:195. doi: 10.1186/s12916-023-02881-z (PMC10210381; doi:10.1186/s12916-023-02881-z)
Supplement: Supplementary file 2 — Additional file 2: Fig. S1. Scatter plots for MR analyses of the causal effect of 11 coagulation factors on endometriosis in UK Biobank. Fig. S2. Plots of leave-one-out analyses for the causal associations in UK Biobank. Fig. S3. Scatter plots for MR analyses of the causal effect of 11 coagulation factors on endometriosis in FinnGen. Fig. S4. Plots of leave-one-out analyses for the causal associations in FinnGen. Fig. S5. Scatter plots for MR analyses of the causal effect of ADAMTS13 and vWF on endometriosis in a meta-analysis. Fig. S6. Plots of leave-one-out analyses for the causal associations in the meta-analysis. [file 12916_2023_2881_MOESM2_ESM.pdf]

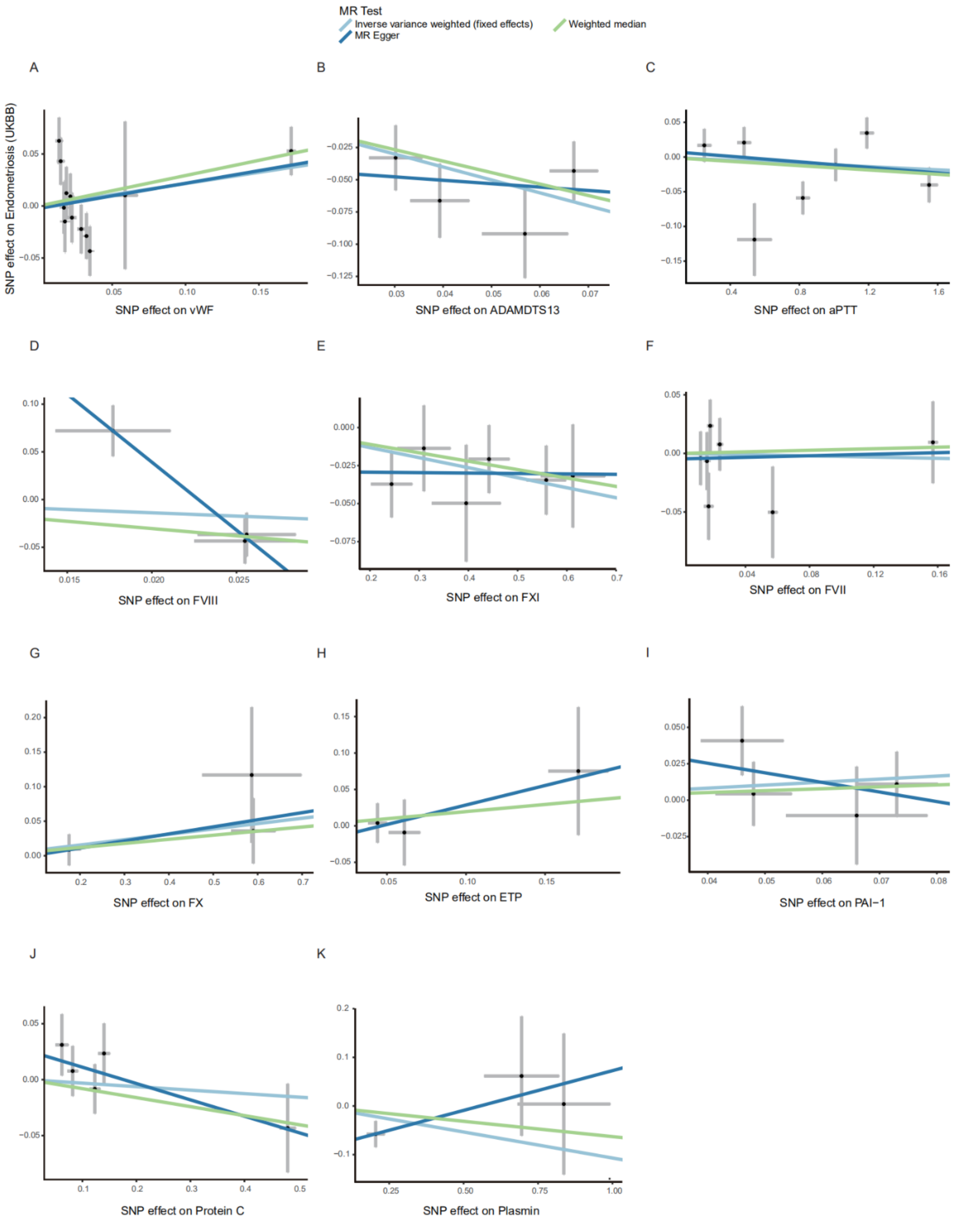

Figure S1. Scatter plots for MR analyses of the causal effect of 11 coagulation factors on endometriosis in UK Biobank.

The MR analyses were conducted using the fixed-effect inverse variance weighted, weighted mean, and MR-Egger. The slop of each line corresponding to the estimated MR effect per method. Error bars around each SNP are 95% confidence intervals. (**A**) vWF, (**B**) ADAMTS13, (**C**) aPTT, (**D**) FVIII, (**E**) FXI, (**F**) FVII, (**G**) FX, (**H**) ETP, (**I**) PAI-1, (**J**) protein C, (**K**) plasmin.

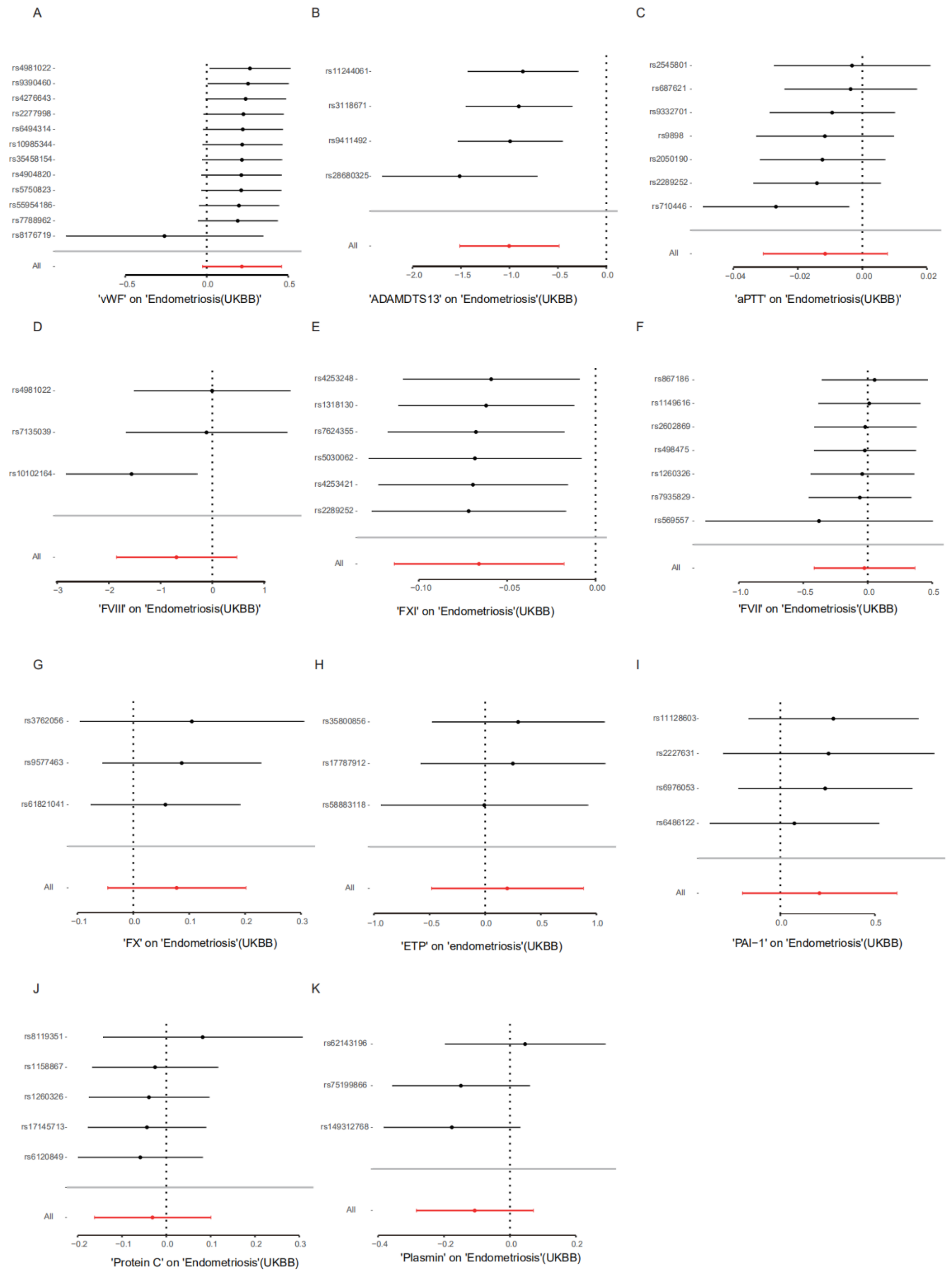

Figure S2. Plots of leave-one-out analyses for the causal associations in UK Biobank. Forest plots depicting causal estimates of 11 coagulation factors on endometriosis by excluding each instrumental variable in turn. The horizontal bars represent beta value and its 95% confidence intervals (CI) of causal estimates. (**A**) vWF, (**B**) ADAMTS13, (**C**) aPTT, (**D**) FVIII, (**E**) FXI, (**F**) FVII, (**G**) FX, (**H**) ETP, (**I**) PAI-1, (**J**) protein C, (**K**) plasmin.

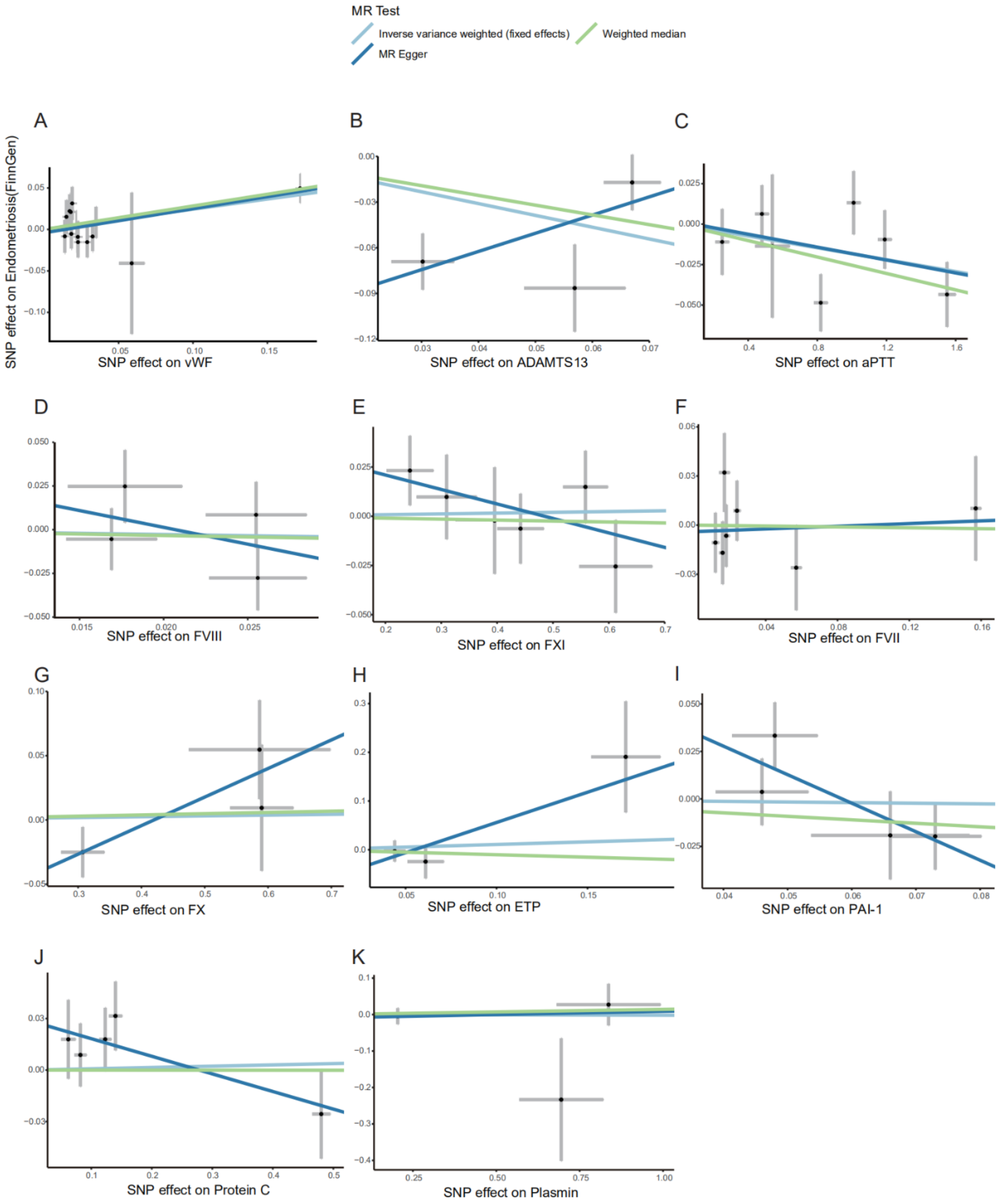

Figure S3. Scatter plots for MR analyses of the causal effect of 11 coagulation factors on endometriosis in FinnGen cohort.

The MR analyses were conducted using the fixed-effect inverse variance weighted, weighted mean, and MR-Egger. The slop of each line corresponding to the estimated MR effect per method. Error bars around each SNP are 95% confidence intervals. (**A**) vWF, (**B**) ADAMTS13, (**C**) aPTT, (**D**) FVIII, (**E**) FXI, (**F**) FVII, (**G**) FX, (**H**) ETP, (**I**) PAI-1, (**J**) protein C, (**K**) plasmin.

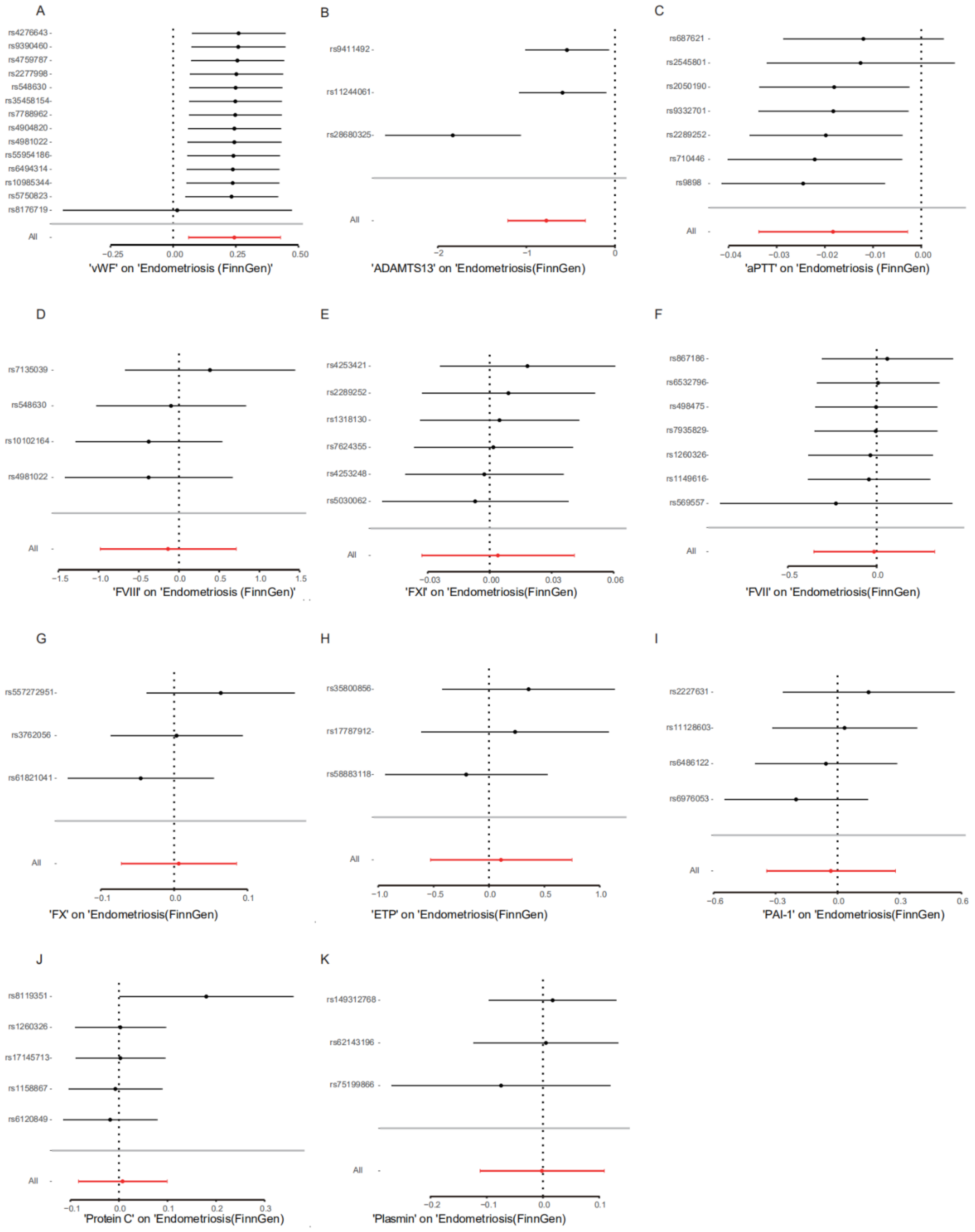

Figure S4. Plots of leave-one-out analyses for the causal associations in FinnGen.

Forest plots depicting causal estimates of 11 coagulation factors on endometriosis by excluding each instrumental variable in turn. The horizontal bars represent beta value and its 95% confidence intervals (CI) of causal estimates. (**A**) vWF, (**B**) ADAMTS13, (**C**) aPTT, (**D**) FVIII, (**E**) FXI, (**F**) FVII, (**G**) FX, (**H**) ETP, (**I**) PAI-1, (**J**) protein C, (**K**) plasmin.

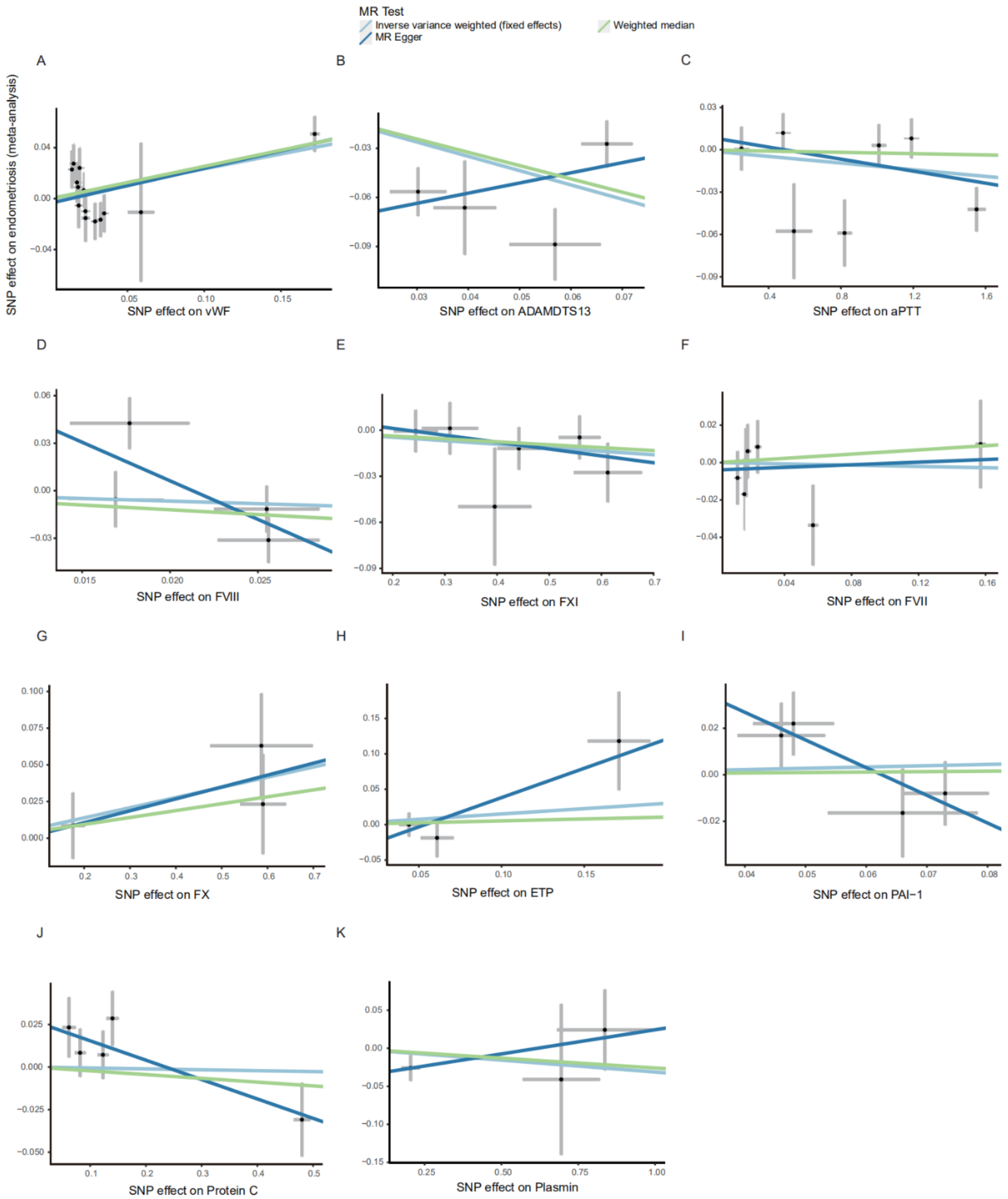

Figure S5. Scatter plots for MR analyses of the causal effect of 11 coagulation factors on endometriosis in a meta-analysis.

The MR analyses were conducted using the fixed-effect inverse variance weighted, weighted mean, and MR-Egger. The slop of each line corresponding to the estimated MR effect per method. Error bars around each SNP are 95% confidence intervals. (**A**) vWF, (**B**) ADAMTS13, (**C**) aPTT, (**D**) FVIII, (**E**) FXI, (**F**) FVII, (**G**) FX, (**H**) ETP, (**I**) PAI-1, (**J**) protein C, (**K**) plasmin.

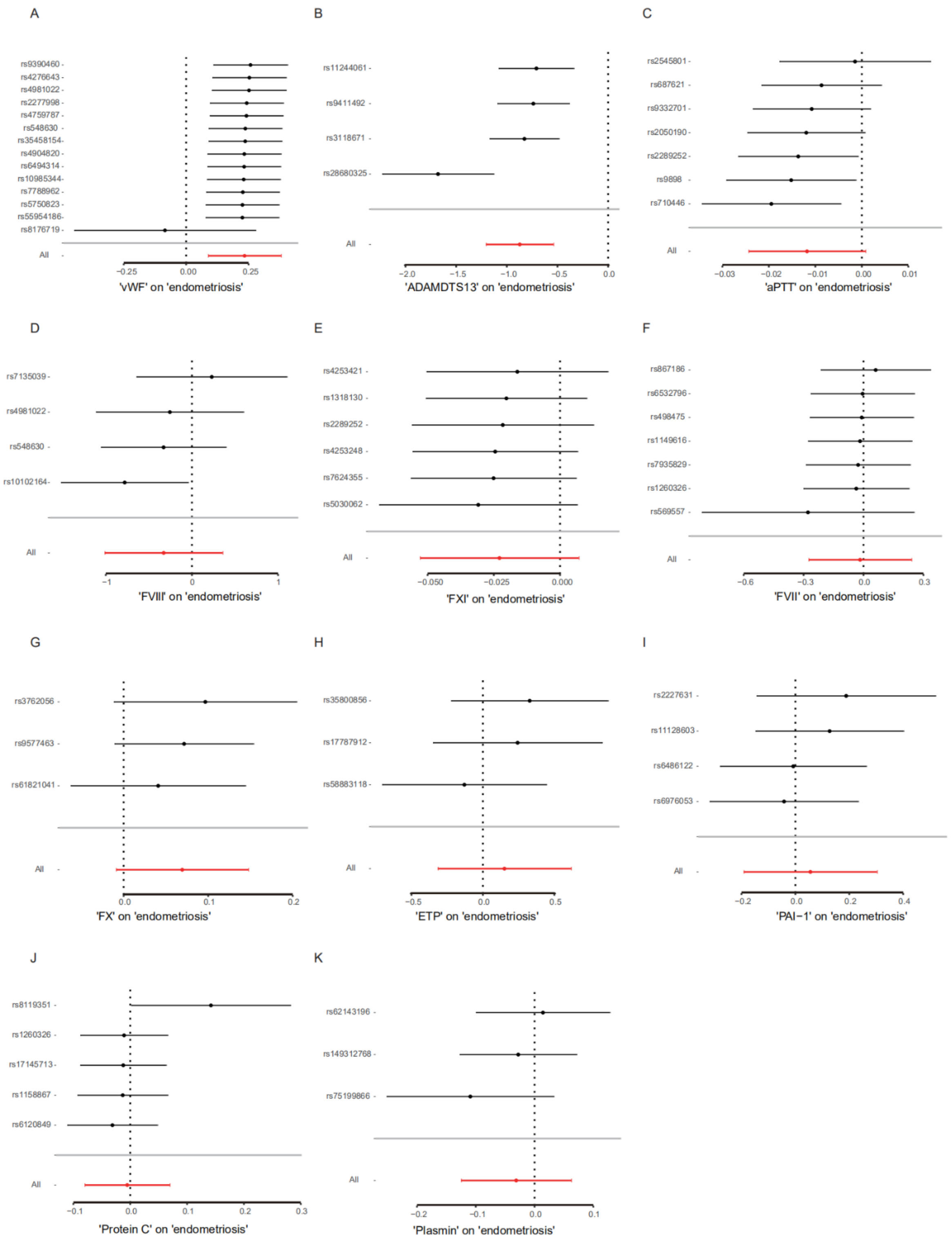

Figure S6. Plots of leave-one-out analyses for the causal associations in the meta-analysis.

Forest plots depicting causal estimates of 11 coagulation factors on endometriosis by excluding each instrumental variable in turn. The horizontal bars represent beta value and its 95% confidence intervals (CI) of causal estimates. (**A**) vWF, (**B**) ADAMTS13, (**C**) aPTT, (**D**)

FVIII, (**E**) FXI, (**F**) FVII, (**G**) FX, (**H**) ETP, (**I**) PAI-1, (**J**) protein C, (**K**) plasmin.
